# Accidental Hypothermia Associated with Intracardiac Thrombi

**DOI:** 10.7759/cureus.4512

**Published:** 2019-04-20

**Authors:** Noel D Torres Acosta, Anas Noman, Ashraf Gohar, Gautam Balakrishnan

**Affiliations:** 1 Internal Medicine, University of Missouri Kansas City School of Medicine, Truman Medical Center, Kansas City, USA; 2 Pulmonary and Critical Care, University of Missouri Kansas City School of Medicine, Truman Medical Center, Kansas City, USA

**Keywords:** accidental hypothermia, intracardiac thrombi, cardiomyopathy

## Abstract

Accidental hypothermia and thrombosis are rarely associated and encountered. A 66-year-old male and 62-year-old male were both admitted with accidental hypothermia. Patient 1 had a rectal temperature of 28.5 °Celcius (C). After 1 day of hospitalization, he developed worsening shortness of breath due to worsening pulmonary edema. Further investigation with echocardiogram showed large left ventricular thrombi as well and global hypokinesis and apical akinesis. Patient 2 had a rectal temperature of 28.5 °C, he was also discovered to have a multifactorial shock. Echocardiogram for shock evaluation showed small apical thrombus as well as global hypokinesis. Hypothermia has been associated with hypocoagulability rather than hypercoagulability secondary to platelet dysfunction and clotting factor enzyme derangements. Moreover, hypothermia has also been associated with myocardial dysfunction that could have predisposed the development of intracardiac thrombi. Further research needs to be done to help better understand these possible association.

## Introduction

Hypothermia is defined as a body temperature below 36 °Celcius (C), further categorized as mild when the temperature is between 32-36 °C, moderate 28-32 °C and severe when the temperature is below 27 °C [1]. Body temperatures below 32 °C are associated with a mortality rate of 23% [2-3]. In the United States, there are about an average of 1301 deaths per year related to hypothermia and 67% of cases occurring in males [4].

Hypothermia has been related to derangements in coagulation and hemostasis. Intracardiac thrombi are usually the result of a low flow state leading to stasis. To our knowledge, these are the first cases in which accidental hypothermia is associated with intracardiac thrombi.

## Case presentation

Case 1 presentation

The patient is a 66-year-old Caucasian male with a past medical history of depression who was brought by the Emergency Medical Services (EMS) after being found down with impaired consciousness in cold temperatures. He was not able to verbalize any complaints.

On physical examination his vital signs were as follows: rectal temperature was 28.5 °C, heart rate 74 beats per minute (bpm), respiratory rate 16 breaths per minute (breaths/min), and blood pressure 130/79 mmHg. At presentation, his Glasgow Coma Scale score was 10/15 (Eye response (E) 4, Verbal response (V) 2, Motor response (M) 4) and was able to protect his airway. Cardiac examination showed rhythmic heart sounds, regular and without murmur; lungs were clear to auscultation. Abdominal examination showed no bowel sounds on auscultation. His skin was extremely cool, pale and moist; peripheral pulses were not palpable. The rest of the examination was unremarkable.

Initial laboratory tests were as: blood gas analysis demonstrated metabolic and respiratory acidosis with the following reading; pH 7.212; partial pressure carbon dioxide (pCO2) 48.5 mmHg; partial pressure of oxygen (pO2) 73.7 mmHg; bicarbonate (HCO3) 19 mmHg on non-rebreather mask at 15 liter per minute (L/min); creatine kinase 811 units per liter (U/L), troponin 0.05 ng/mL, lactic acid 2.4 mmol/L, international normalized ratio (INR) 1.5, activated partial thromboplastin time (APTT) 39.6 seconds (sec), prothrombin time (PT) 17.3 sec, aspartate aminotransferase (AST) 56 U/L, alanine aminotransferase (ALT) 47 U/L, glucose 145 mg/dL. Urine drug screening was negative to any substances and alcohol level was <10 mg/dL. Initial electrocardiogram (EKG) showed a wide QRS rhythm, rightward axis, non-specific intra-ventricular conduction block. Initial chest x-ray showed diffuse bilateral heterogenous opacities consistent with pulmonary edema.

**Figure 1:**
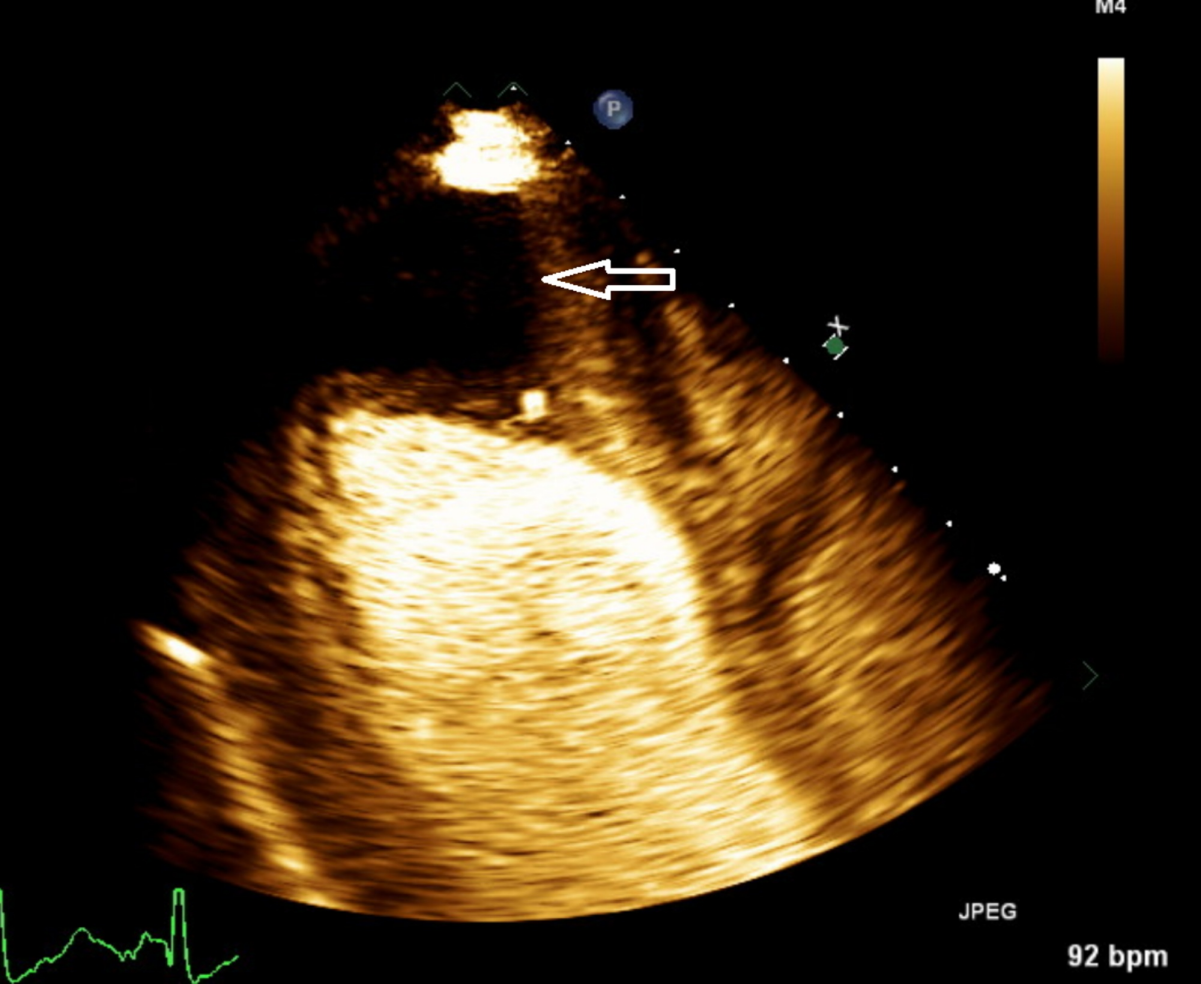
Patient 1 Echocardiogram

The patient was diagnosed with severe hypothermia and was subsequently started on slow controlled re-warming therapy with Arctic Sun (Bard temperature management system) with success returning to a normal temperature within the next day with an improvement of his mentation. The next day he developed shortness of breath and increased oxygen requirements, chest X-ray was done and showed worsening pulmonary edema, pro-brain natriuretic peptide (pro-BNP) was 2,776 pg/mL and troponins trended up to 0.10 ng/mL. He subsequently had echocardiogram which showed severely reduced left ventricular systolic function, with estimated ejection fraction (EF) <20%; severe global hypokinesis and akinetic apex occupied by 2 layers of large thrombi (3.7 x 2.5 cm and 1.2 x 4.8 cm) (Figure 1).

Outcome and Follow-up

The patient was started on anticoagulation with heparin and transitioned to warfarin. After 7 days of anticoagulation, the patient developed large gastrointestinal bleeding leading hypovolemic shock secondary to a bleeding duodenal ulcer and anticoagulation was stopped. Through the rest of the hospital stay, the patient had several episodes of gastrointestinal bleeding and he made the decision not to continue with more treatment for gastrointestinal bleeding and died after 18 days of hospitalization secondary to hypovolemic shock.

Case 2 presentation

The patient is a 62-year-old African American male with an unknown past medical history who was brought by EMS after being found down at his home without heat by his neighbor.

On physical examination, his vital signs were: rectal temperature was 28.5 °C, heart rate 94 bpm, respiratory rate 22 breaths/min, and blood pressure 170/100 mmHg. On presentation, his Glasgow Coma Scale was 8 (E4 V1 M3). He was found to be non-responding verbally, pupils were reactive to light, lungs clear to auscultation, the heart had regular rate and rhythm. He was intubated in the emergency department.

Initial laboratory with blood gas analysis showed a metabolic and respiratory acidosis with the following pH 7.103; pCO2 65.6 mmHg; pO2 514.9 mmHg; HCO3 20.0 mmHg on the following ventilator settings: tidal volume 450 mL, respiratory rate 18 breaths/min, fraction of inspired oxygen (FiO2) 100% and positive end-expiratory pressure (PEEP) of 5 cmH2O. Other laboratory findings were the following: sodium 165 mmol/L, potassium 3.0 mmol/L, chloride 122 mmol/L, carbon dioxide (CO2) 18 mmol/L, glucose 182 mg/dL, blood urea nitrogen (BUN) 86 mg/dL, creatinine 1.83 mg/dL, AST 92 U/L, ALT 56 U/L, lipase 300 U/L, creatinine kinase 1,661 U/L, troponin 0.02 ng/mL, white blood cell count (WBC) 29.50 x 10^3 ^cmm, hemoglobin 5.4 g/dL, platelets 225 x 10^3 ^cmm, PT 17 sec, INR 1.5, APTT 36.4 sec, lactic acid 6.3 mmol/L. Urine drug screening was negative for any substances and alcohol level was <10 mg/dL. Initial EKG showed atrial fibrillation with premature ventricular complexes, left axis deviation, lateral injury pattern. Initial chest x-ray showed diffuse opacities on bilateral lung fields consistent with aspiration pneumonitis or infection. 

**Figure 2:**
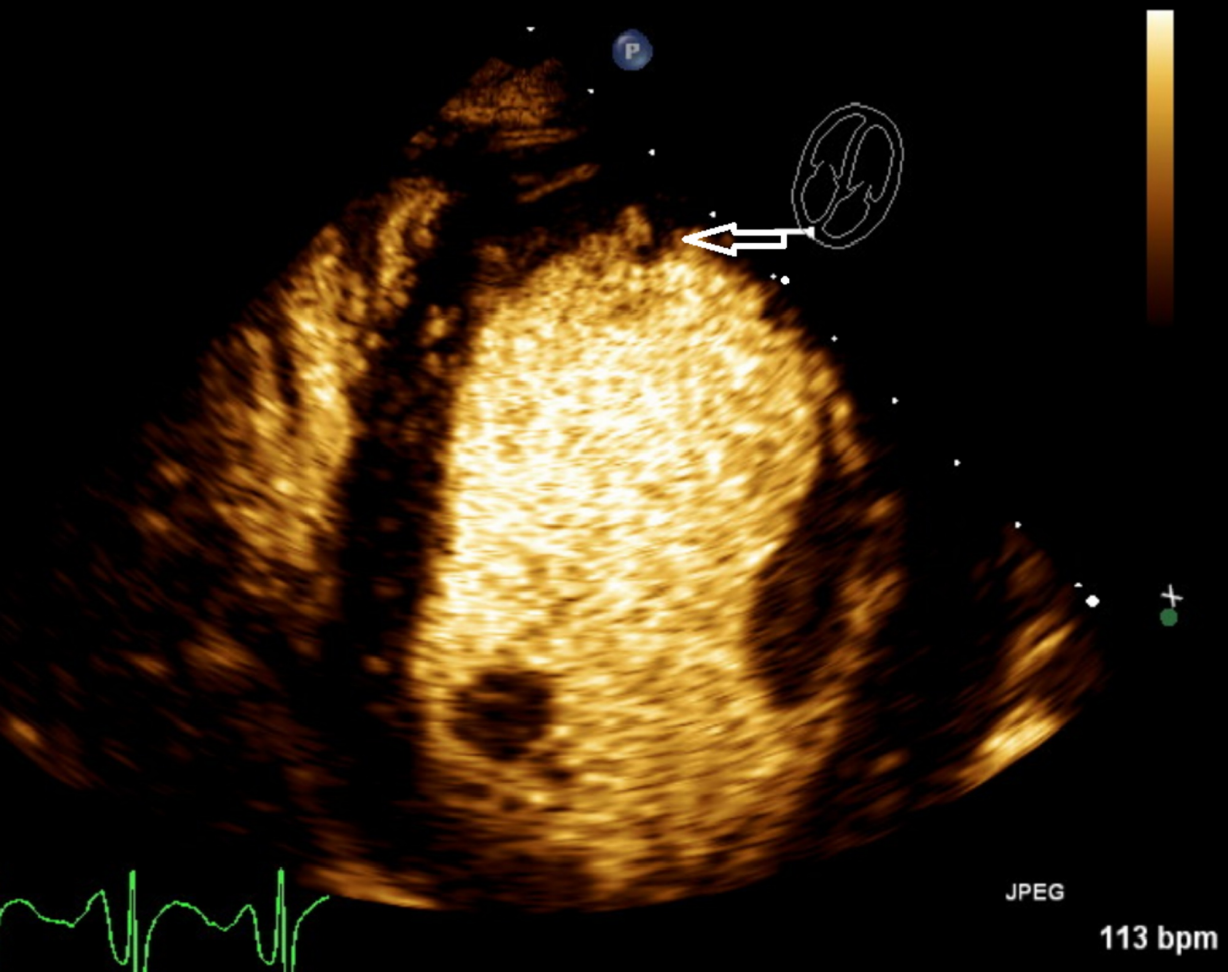
Patient 2 Echocardiogram

The patient was admitted to the Intensive Care Unit (ICU) for rewarming with IV fluids and Bair Hugger (3M temperature management system). He developed multifactorial shock and was started on vasopressors, blood transfusion, and antibiotics. An echocardiogram was done 2 days after admission for shock investigation which demonstrated moderate left ventricular hypertrophy, anteroseptal and anterior akinesis with global hypokinesis, ejection fraction 25% with suspicion of apical thrombus and left atrial enlargement (Figure 2).

Outcome and Follow-up

The patient was later started on anticoagulation after anemia resolved but anticoagulation was later held as he developed a psoas hematoma. He had 2 subsequent echocardiograms including a transesophageal echocardiogram during hospitalization that were concerning for apical thrombus. The patient was later discharged to a nursing home facility after 80 days of hospitalization with multiple complications.

## Discussion

Hypothermia has been associated with derangements on platelet function and coagulation. In cases of mild to moderate hypothermia platelet function is affected but in severe hypothermia clotting factor enzyme activity is affected as well [3]. The optimal temperature to maintain balanced hemostasis is 37.5 C [5]. Whelihan et al. found on in vitro experimentation that severe hypothermia leads to delay in thrombin generation secondary to delayed thrombin initiation; reduction on initial fibrinogen consumption and factor XIII activation [3]. Moreover, hypothermia affects global hemostasis and decreases the rate of clot formation and lysis without affecting the clot strength. Several studies in patients undergoing cooling after cardiac arrest or as part of laboratory studies have shown impairment of platelet function, and hypocoagulability [5]. In the setting of hypothermia, intracardiac thrombus as a direct consequence has not been described in the literature. The majority of the literature presented has shown that hypothermia is associated with hypocoagulability rather than with hypercoagulability and thrombosis. Some authors have found some associations of catheter-related thrombosis in the setting of hypothermia but endovascular catheters are on itself a risk factor for thrombosis [6]. However, there have been some data suggesting that hypothermia can cause impairment of prostacyclin PGI2 synthesis which can result in platelet activation and thrombosis and could have been the underlying mechanism in these patients [1].

Another mechanism that could have led to the left ventricle thrombosis is hypothermia induced cardiomyopathy. Cardiovascular changes seen in hypothermia are dependent on the degree of hypothermia. Initially, there is tachycardia and vasoconstriction with an increment in cardiac output; as hypothermia progresses patients develop bradycardia, hypotension with a subsequent decrease in cardiac output as well as atrial and ventricular arrhythmias [1,7]. When temperatures fall below 20 C, asystole is seen [8]. Research has shown that hypothermia on itself could be a factor for contractile dysfunction [9]. The pathophysiology of hypothermia-induced cardiac dysfunction was explained by experimental research which showed changed in cardiac myofilament calcium (Ca2+) sensitivity and/or in the phosphorylation level of cardiac troponin I (cTnI), thus contributing to reduced contractility [9]. Thus, intracardiac thrombi can develop as a result of cardiac dysfunction or asystole through a low flow state. This low flow state leading to stasis and thrombus is also found in left ventricle thrombi secondary to myocardial infarction (MI). The risk factors in these cases would usually depend on the size of the infarct, severe apical akinesias or dyskinesis, aneurysms, and anterior MI [10]. Recently the incidence of left ventricle (LV) thrombus has declined due to improved treatment of acute coronary syndromes in the acute phase [10].

Moreover, rewarming following hypothermia has been shown to induce myocardial failure by reducing the cardiac output and stroke volume [11]. Also, there is an overload in intracellular myocardial tissue calcium content that may contribute to heart failure [11]. Experimentation in animal models has shown that rewarming following hypothermia resulted in systolic dysfunction associated with an increase in troponin T that may indicate cardiac tissue degradation [12]. The above mechanism could be another explanation for these patients presentation as heart failure could be the culprit for a procoagulable state. 

## Conclusions

In short, both of our patients presented with severe hypothermia and were found to have severe cardiomyopathy and left ventricular thrombi. Hypothermia has been associated with decreased cardiac function and to lead to global hypokinesis. There has been also some data suggesting that hypothermia is a procoagulant state instead of anticoagulant. Further research needs to be done to help better understand this possible association.
